# Supplementary material for: A Critical View of the Application of the APEX Software (Aqueous Photochemistry of Environmentally-Occurring Xenobiotics) to Predict Photoreaction Kinetics in Surface Freshwaters
Source: Molecules. 2019 Dec 18;25(1):9. doi: 10.3390/molecules25010009 (PMC7017383; doi:10.3390/molecules25010009)
Supplement: Supplementary file 1 [file molecules-25-00009-s001.zip › 3.2.4_gcc-4.4.0.zip!/3.2.4_gcc-4.4.0/3.2.4_gcc-4.4.0/doc/octave/html/interpreter/Differential-Equations.html]

Differential Equations - Untitled


Next: Optimization,
Previous: Numerical Integration,
Up: Top


---

## 23 Differential Equations

Octave has built-in functions for solving ordinary differential equations,
and differential-algebraic equations.
All solvers are based on reliable ODE routines written in Fortran.

- Ordinary Differential Equations- Differential-Algebraic Equations
